# An integrated digital PCR system with high universality and low cost for nucleic acid detection

**DOI:** 10.3389/fbioe.2022.947895

**Published:** 2022-08-19

**Authors:** Kangning Wang, Bin Li, Yu Guo, Yanqi Wu, Yan Li, Wenming Wu

**Affiliations:** ^1^ Institute of Biological and Medical Engineering, Guangdong Academy of Sciences, Guangzhou, China; ^2^ Institute of Microbiology Chinese Academy of Sciences, Beijing, China; ^3^ School of Mechanical and Electrical Engineering, Guangdong University of Technology, Guangzhou, China; ^4^ State Key Laboratory of Quality Research in Chinese Medicine, Macau University of Science and Technology, Taipa, China

**Keywords:** SARS-CoV-2 virus, digital PCR, dPCR chip, fluorescence detection, absolute quantification

## Abstract

Digital PCR is the most advanced PCR technology. However, due to the high price of the digital PCR analysis instrument, this powerful nucleic acid detection technology is still difficult to be popularized in the general biochemistry laboratory. Moreover, one of the biggest disadvantages of commercial digital PCR systems is the poor versatility of reagents: each instrument can only be used for a few customized kits. Herein, we built a low-cost digital PCR system. The system only relies on low-cost traditional flat-panel PCR equipment to provide temperature conditions for commercial dPCR chips, and the self-made fluorescence detection system is designed and optically optimized to meet a wide range of reagent requirements. More importantly, our system not only has a low cost (<8000 US dollars) but also has a much higher universality for nucleic acid detection reagents than the traditional commercial digital PCR system. In this study, several samples were tested. The genes used in the experiment were plasmids containing UPE-1a fragment, TP53 reference DNA, hepatitis B virus DNA, leukemia sample, SARS-COV-2 DNA, and SARS-COV-2 RNA. Under the condition that DNA can be amplified normally, the function of the dPCR system can be realized with simpler and low-price equipment. Some DNA cannot be detected by using the commercial dPCR system because of the special formula when it is configured as the reaction solution, but these DNA fluorescence signals can be clearly detected by our system, and the concentration can be calculated. Our system is more applicable than the commercial dPCR system to form a new dPCR system that is smaller and more widely applicable than commercially available machinery.

## Introduction

Polymerase chain reaction (PCR), a method for enzymatic synthesis of specific DNA *in vitro*, has been proposed for 20 years. PCR reaction procedure consists of denaturation, annealing, and extension for one cycle. After several cycles, the target DNA can be massively amplified in a short time. To date, PCR has developed into the conventional key technology in the field of molecular biology, greatly promoting the development of various fields of life sciences. Especially in the late 1990s, the quantitative real-time PCR (qPCR) technology (Ginzinger, 2002; Arya et al., 2005; Ma et al., 2013) and related products from *in vitro* synthesis of quantitative and qualitative/detection techniques developed into a highly sensitive, specific, and accurate quantitative analysis technology.

Considering the rapid development over the past decades, the qPCR technology has been used for the diagnosis of many diseases (Coulson et al., 2008; Taniguchi et al., 2009; van Eijk et al., 2011; Song et al., 2012; Moreira et al., 2013). However, there are many factors that affect the amplification efficiency during PCR amplification. There is no guarantee that the amplification efficiency will remain constant during the reaction. In other words, the amplification efficiency may be different between the work samples and the standard samples. This results in the fact that the cyclic threshold (CT) on which quantitative analysis depends is not constant. Therefore, the quantification of the qPCR is only “relative quantification,” and its accuracy and reproducibility still cannot meet the requirements of quantitative analysis of molecular biology.

At the end of the 20th century, Vogelstein et al. proposed the concept of digital PCR (dPCR) by dividing a sample into tens to tens of thousands of different reaction units, where each unit contained one or more copies of the target molecule (DNA template) (Sanders et al., 2011; Whale et al., 2012; Hudecova, 2015; Schuler et al., 2016; Sreejith et al., 2018). The target molecule is subjected to PCR amplification in each reaction unit, and the fluorescence signal of each reaction unit is statistically analyzed after the amplification. Unlike qPCR, digital PCR does not depend on CT values, so it is not affected by the amplification efficiency. At the end of the amplification, the average concentration (content) of each reaction unit can be calculated by direct counting or employing the Poisson distribution formula. Under the control of less than 5%, digital PCR can realize absolute quantitative analysis without standard samples and curves. Droplet dPCR (Pinheiro et al., 2011; Tian et al., 2015; Gou et al., 2018; Song et al., 2018) is actually a miniaturized traditional PCR amplification. In this technique, the reaction solution is uniformly introduced into the reaction chamber or through hole, and each chamber or through hole is scanned using microfluidic chip technology and fluorescence signal. The method is similar to the detection method of the gene chip to calculate the content of the target sequence. However, the droplet dPCR technology is widely used due to its maturity.

During the past 10 years, different companies have explored different technologies and methods to realize digital PCR automation. At present, the available digital platforms are mainly different in the number of liquid separations, droplet generation methods, and special equipment required. BioMark^TM^HD system provides a matrix dedicated to digital integrated fluid circuits (IFCs), which can distribute samples in multiple separate reaction chambers. QuantStudio 3D system uses a silicon chip composed of a single reaction hole arranged in a certain order, which can distribute samples according to its matrix. CONSTELLATION^®^ digital PCR system uses a microporous plate using sealed compression axis; the micropipette separates the sample liquid channel into a separate microfluidic chamber. Other digital PCR platforms such as QX200^TM^DropletDigital^TM^PCR and RainDropplus™ digital PCR systems use oil-in-water emulsification to partition. The water phase is composed of primers, probes, or fluorescent dyes and mixed into super premixes; the samples and mineral oil are put into specially designed scaffolds, the droplet generator uses microfluidics to generate pressure, and the water phase and oil phase are sucked into the output channel. In this process, droplets are formed, and each droplet is read in a specific droplet reader one by one. The Naica system from Stilla Technologie™ company combines the array and emulsification methods. In this system, the sample passes through the channel of the chip and forms droplets inside the chip, which becomes an ideal digital PCR technology platform.

At present, dPCR technology, also regarded as liquid biopsy, is mainly used for detecting trace DNA. Its clinical applications include tumor fluid biopsy, (Sacher et al., 2016; Conteduca et al., 2017; van Ginkel et al., 2017; Wang et al., 2018), noninvasive prenatal screening (Tan et al., 2019), early diagnosis of infectious diseases, (Cao et al., 2019), transplant rejection monitoring (Beck et al., 2013) and other fields. The detection object of tumor liquid biopsy is the circulating tumor DNA (ctDNA) in blood or other body fluids, which not only realizes non-invasive diagnosis and detection but also can monitor the course of the disease and guide the treatment. dPCR is a powerful tool to detect low abundance mutations. At present, ctDNA detection technology based on dPCR has achieved encouraging results in the research of nonsmall cell lung cancer, (Sacher et al., 2016), prostate cancer, (Conteduca et al., 2017), head and neck squamous cell carcinoma, and other tumors (HNSCC) (van Ginkel et al., 2017).

In this study, we realized the function of the dPCR system by using the simple traditional plane PCR instrument and detection system, which made the whole system simpler, smaller, and cheaper. Moreover, the detection range of commercial dPCR is limited due to the need for specific reagent formulation. But the system is more user-friendly and accurate.

## Materials and method

### Reagents

**FIGURE 1:**
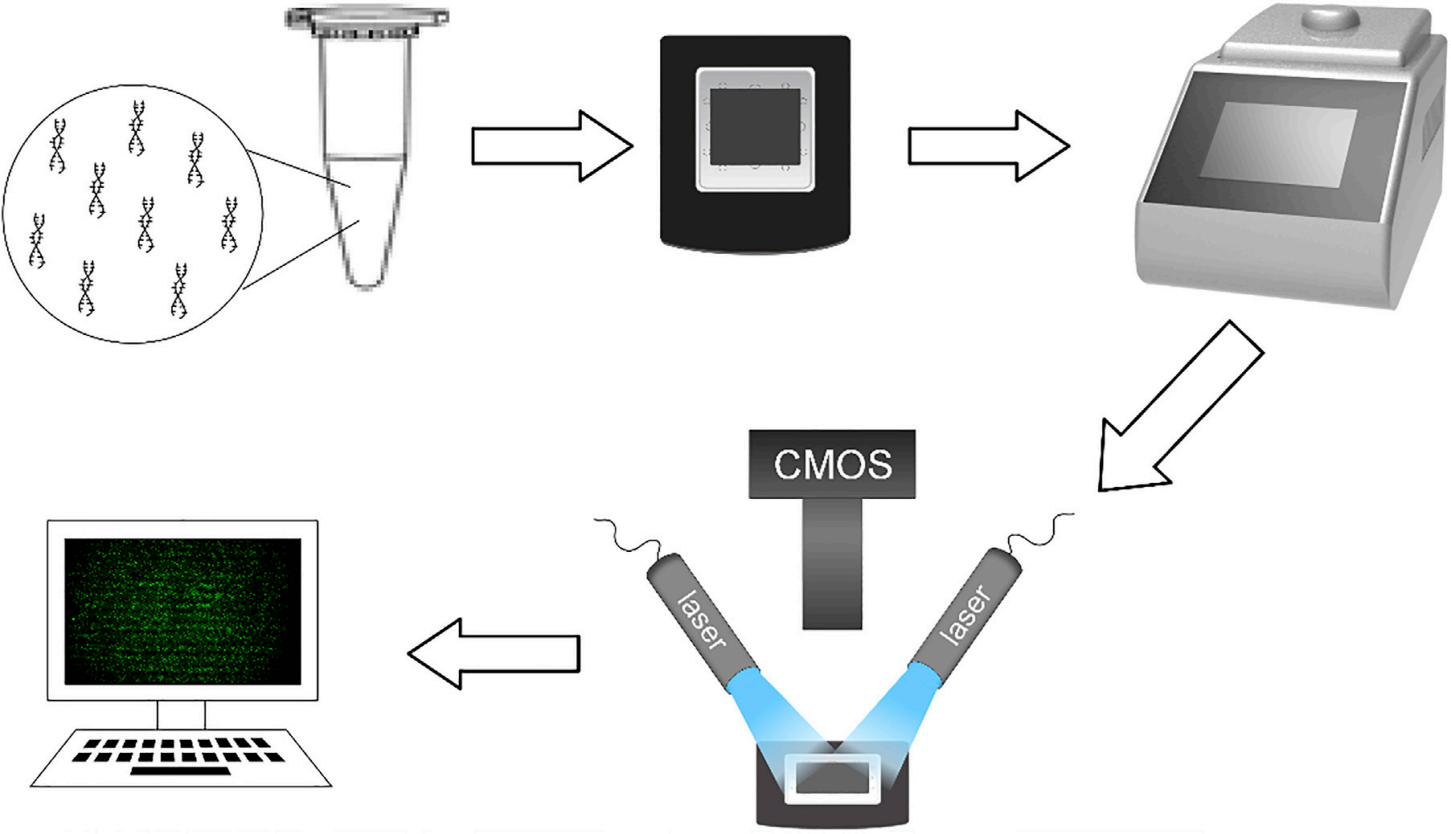
Flow chart of dPCR system.

In this study, several samples were tested. The genes used in the experiment were plasmids containing SARS-COV-2 DNA fragment (N gene) provided by Sangon Biotech (Shanghai) Co., Ltd. (abbreviated as SARS-COV-2 DNA in the following description), plasmids containing UPE-1a fragment (abbreviated as UPE DNA in the following description), RNA sample of SARS-COV-2 (abbreviated as SARS-COV-2 RNA in subsequent narratives) provided by Wuhan Institute of Virology, Chinese Academy Of Sciences, TP53 reference DNA (abbreviated as TP53 DNA in the following description) provided by RainSure biology company, hepatitis B virus DNA (abbreviated as HBV DNA in the following description) provided by Northeast Pharmaceutical Company, and the genetic sample of a leukemia patient provided by First Bethune Hospital of Jilin University (abbreviated as leukemia sample in the following description). 2x ddPCR Supermix for probes (NO dUTP) was produced from Bio Rad. Before the experiment, the sample was configured as a mixture of reaction solution according to the formula in the instruction manual. Some of the mixture was coated in the dPCR chip as the experimental group. After all the preparations, the dPCR chips with DNA samples distributed were placed in the conventional planar PCR (BIOER Gene Touch TC-EA) for amplification (Figure 1). A part of the reaction solution was tested by the commercial dPCR system (RainSure), and the results were used as the standard to measure the success of the experiment.

### Digital Polymerase chain reaction chip

Droplet dPCR does not depend on the cyclic threshold to determine the number of targets. Therefore, the difference in PCR efficiency between biological samples will not affect the quantitative results.

Although the principle of dPCR technology is not complicated, it is difficult to break through in the early stage of sample distribution in the first step, and the quantity and uniformity of distribution are very low, which greatly limits the development of dPCR. Until the last few years, with the emergence of water-in-oil emulsified droplets, integrated fluidic circuit (IFC), nanofabrication, and other technologies, dPCR technology has finally broken through the technical bottleneck and successfully commercialized. Among them, the droplet dPCR technology is represented by Life Technology’s QuantStudio 3D system. Using high-density nanoliter fluidic chip technology, a standard PCR reaction is evenly distributed to up to 20,000 individual reaction holes in the chip. Each reaction well contains or does not contain one or more copies of the target molecule (DNA template) so as to realize “single molecule template PCR amplification.” The fluorescence signal of each reaction chamber is scanned by a method similarly to a gene chip to calculate the content of the target sequence. Currently, each chip is integrated with 10,000–40,000 (BioMark HD) reaction chambers or 20,000 (QuantStudio 3D) reaction well. However, the high price of PCR machines has deterred many organizations that need them from buying them.

In this study, only the chip of QuantStudio 3D system was selected as the reflecting container, and the denaturation, renaturation, and elongation temperatures required for polymerase chain reaction were provided in other cheaper devices.

Twenty thousand reaction wells of QuantStudio^®^ 3D Digital PCR 20K chip are fabricated on a 10 square millimeter silicon wafer to make dPCR chips. Each well is a regular hexagon with a diagonal length of 60 µm. Each reaction well is isolated from its neighbors. Fixed reaction volume minimizes upfront sample manipulation.

### Image acquisition and processing

In our former fluorescence detection system, the high-power blue LED with a power of 3 W was used as the fluorescence excitation light source, and a narrow-band filter with a central wavelength of 480 nm was installed in front of the excitation light source to ensure that it could excite the fluorescence, but it would not generate redundant noise. A 20-megapixel CMOS (complementary metal oxide semiconductor) was selected as the fluorescence receiving device. An optical lens and a narrow-band filter with a central wavelength of 520 nm were installed in the front-end of the CMOS to receive the fluorescence signal, and the real-time image was displayed on the PC connected with it. But in the experiment, we found that LED cannot provide enough light energy for the microdroplets in the chip, which led to the fact that the fluorescence signal could not be detected by CMOS in the initial experiment. In order to solve the abovementioned problems, we chose higher energy and more concentrated laser (Anford T850AD1670GD-P488 60 mW) as the light source for excitation fluorescence. The advantages of laser, such as small volume, light weight, good monochromaticity, high energy concentration, and reliability, were just suitable for our requirements of excitation light source. The lens was used to irradiate the dPCR chip as evenly as possible. Therefore, two small lasers with a central wavelength of 488 nm were selected as excitation sources and placed symmetrically on both sides of the chip. CMOS was placed in the center of two small lasers, which was convenient to collect the fluorescence information in the chip. Of course, for weak fluorescence detection, the appropriate optical barrel is indispensable. The optical tube received the fluorescence as much as possible and projected it on the sensitive element of the receiver. At the same time, it was important for enlarging the chip to be observed to an appropriate size.

### Concentration calculation

Digital PCR technology is an absolute quantitative technology of nucleic acid molecules. The principle is to distribute the PCR reaction system to a large number of micro reactors. Each micro reactor contains or does not contain one or more copies of the target nucleic acid molecules (DNA templates) for “single molecule template” PCR amplification. At the end of amplification, the number of positive reaction units (judged by terminal fluorescence signal) and the number of copies of target genes in the original samples were calculated by statistical method. The workflow mainly includes four steps (as shown in the figure below): PCR/RT-PCR reaction premixing sample DNA/RNA, reaction premixing liquid dispersion or division, PCR amplification, fluorescence signal acquisition, and data analysis. Digital PCR disperses the sample DNA into 25,000–30,000 microdrops so that each microdrop does not contain or contains one or more copies of the target molecule (DNA template). All microdrops are randomly spread in the sapphire chip in the form of a 2D array. After amplification, the number of template copies in the original sample was calculated by counting the number of positive reaction holes. Because the target DNA molecules are randomly distributed in the positive reaction units and directly count and count the positive reaction units, it is not the true copy number of the target DNA molecules. Each reaction unit may contain two or more target molecules. We used Poisson probability distribution Eq. 1 to calculate.

$$\text{p}=\frac{e^{-\lambda }}{k!}\lambda^{k},\text{λ}=0,\;1,\;2\ldots ,$$ (1)

In the above Eq. 1, λ is the average number of copies of starting DNA molecules contained in each reaction unit, and *p* is the probability of each reaction unit containing k copy target molecules under certain λ conditions. λ is determined by the dilution coefficient m (or the number of zones) of the sample solution, i.e., λ = cm, where c is the original copy number of the sample. When k = 0, i.e., without the target DNA molecule, *p* is the ratio between the number of reaction units without fluorescent signal and the total number of reaction units, i.e., the ratio of negative reaction units. Eq. 1 can be simplified as Eq. 2:

$$\text{p}=e^{-\lambda }\left(k>0,\;\lambda \right)=1-e^{-\lambda }\left(k=0,\;\lambda \right)=1-e^{-cm}$$ (2)

Through the end-point method, the total number of reaction units n and the number of positive reaction units f with fluorescence signal can be reached, so the proportion of negative reaction units is Eq. 3:

$$\text{p}=\frac{n-f}{n}$$ (3)

Taking the logarithm with e as the base on both sides of the abovestated formula, the following equation is obtained:

$$\text{cm}=-\text{ln}\left(1-\frac{f}{n}\right)$$ (4)

When using the method of digital PCR to carry out the absolute quantitative analysis of nucleic acid, only through the proportion of negative reaction units and the dilution coefficient (or partition number) of samples, the average number of nucleic acid copies of reaction units can be determined, thus realizing the accurate quantitative analysis of DNA.

## Results and discussion

In order to prove the applicability of the proposed dPCR device, we designed multiple sets of control experiments to analyze the gene fragments by using dPCR. First, we prepared UPE-1a plasmid DNA from Wuhan Virus Research Institute and a TP53 reference DNA sample from Swiss Biosciences at a concentration. The dPCR reaction mixture was prepared for each panel. The 20 µl of reaction mixture containing UPE-1a DNA included the following: 10 µl of 2x ddPCR SuperMix (No dUTP), 1 µl of Mntant FAM probe, 2 µl of reference STD DNA, and 7 µl of sterile double distilled water. The reaction mixture containing TP53 reference DNA included the following: 10 µl of 2x ddPCR SuperMix for probes (NO dUTP), 0.75 µl of upEF, 0.75 µl of upER, 0.5 µl of upEP (FAM), 1 µl of template solution, and 7 µl of sterile double distilled water. The 20 µl of reaction mixture containing the leukemia sample included the following: 10 µl of 2x ddPCR SuperMix (No dUTP), 2 µl of a mixture of primer and probe, 1 µl of leukemia sample, and 7 µl of sterile double distilled water. The 20 µl of reaction mixture containing SARS-COV-2 DNA included the following: 10 µl of 2x ddPCR SuperMix (No dUTP), 1 µl of a mixture of primer and probe, 1 µl of SARS-COV-2 DNA, and 8 µl of sterile double distilled water. The 50 µl of reaction mixture containing SARS-COV-2 RNA included the following: 10 µl of 5x One-step RT-PCR buffer, 5 µl of Solution Ι (10x), 2 µl of Abstart Taq (with dNTP), 2.5 µl of a mixture of primer and probe, 20 µl of SARS-COV-2 RNA, and 10.5 µl of sterile double distilled water. In the experiment, 15 µl of the mixture was placed on a special blade, the mixture was evenly applied to the chip, and finally, the sealing oil was injected (immersion fluid) to prevent evaporation of the mixture during heating.

The thermal-cycling scheme of UPE DNA, TP53 reference DNA, leukemia sample, and SARS-COV-2 DNA starts from the pre-denaturation of 95°C for 10 min, the denaturation temperature is 94°C, lasting for 30 s, and the renaturation and extension temperature is 55°C, maintaining for 60 sb with 40 cycles. SARS-COV-2 RNA added a reverse transcription process at 55°C for 30 min. HBV DNA is replicated by two steps: 94°C of denaturation temperature lasts for 10 s, 60°C of renaturation and extension temperature lasts for 30 s. The experiment lasts 40 cycles. In order to make the PCR reaction more stable, the temperature change rate the of *in-situ* PCR instrument was set to 0.5°C/s. During the experiment, we found that although the excitation light source was replaced by a small laser, the cover of the dPCR chip would still block part of the light energy irradiated on the droplet. Therefore, we increased the number of lasers to two and placed the lasers symmetrically on both sides to avoid weak light energy and uneven distribution caused by irradiation on one side while not interfering with image acquisition.

The prepared reaction liquid mixture (FAM channel) was evenly coated on the dPCR chip, the sealing cover was covered, and the sealing oil was injected. Then, the chip was placed in the PCR machine, and the thermal cycle was set as described earlier. The heat cover temperature was set to 80°C. After thermal cycling, the dPCR chip was placed in the fluorescence observation system to observe the fluorescence.

**FIGURE 2:**
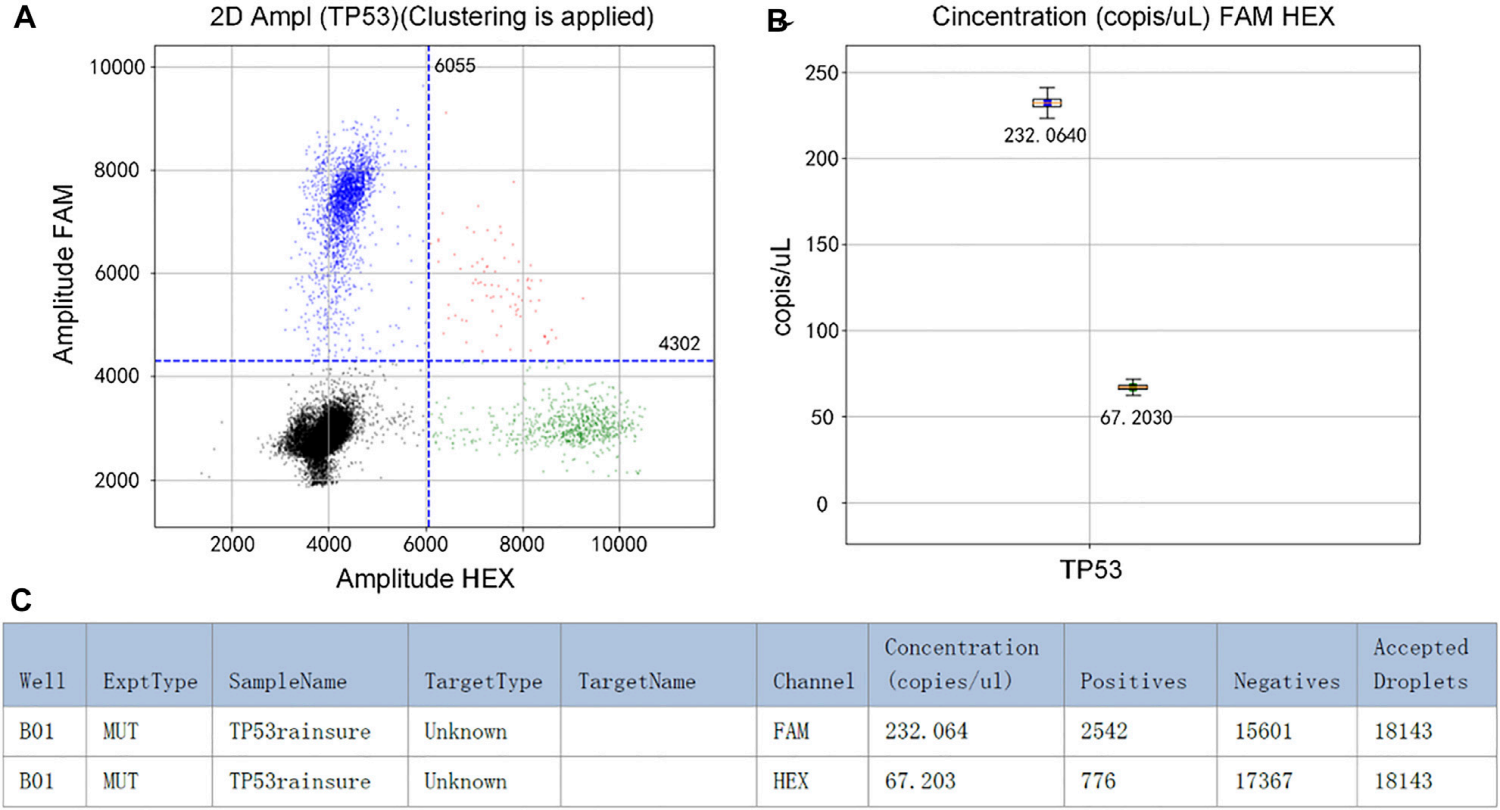
**(A)** Scatter plot by RainSure dPCR; **(B)** Initial DNA concentration calculated by RainSure dPCR; **(C)** Microdroplet data derived by RainSure dPCR.

The initial concentration of TP53 DNA was 10^4^ copies/µl. In the experiment, the mixture was divided into two parts. One of them was detected by RainSure dPCR as the control group. The other was coated on the dPCR chip as the experimental group and placed in the plane PCR instrument for amplification. If the results of the two methods are similar, it will be proved that our method can replace the expensive dPCR equipment very well. Figure 2A shows that the fluorescence could be easily identified after amplification. In the FAM channel, the DNA concentration was 232.0640 copies/µl (Figure 2B). Details of the droplet are given in Figure 2C.

**FIGURE 3:**
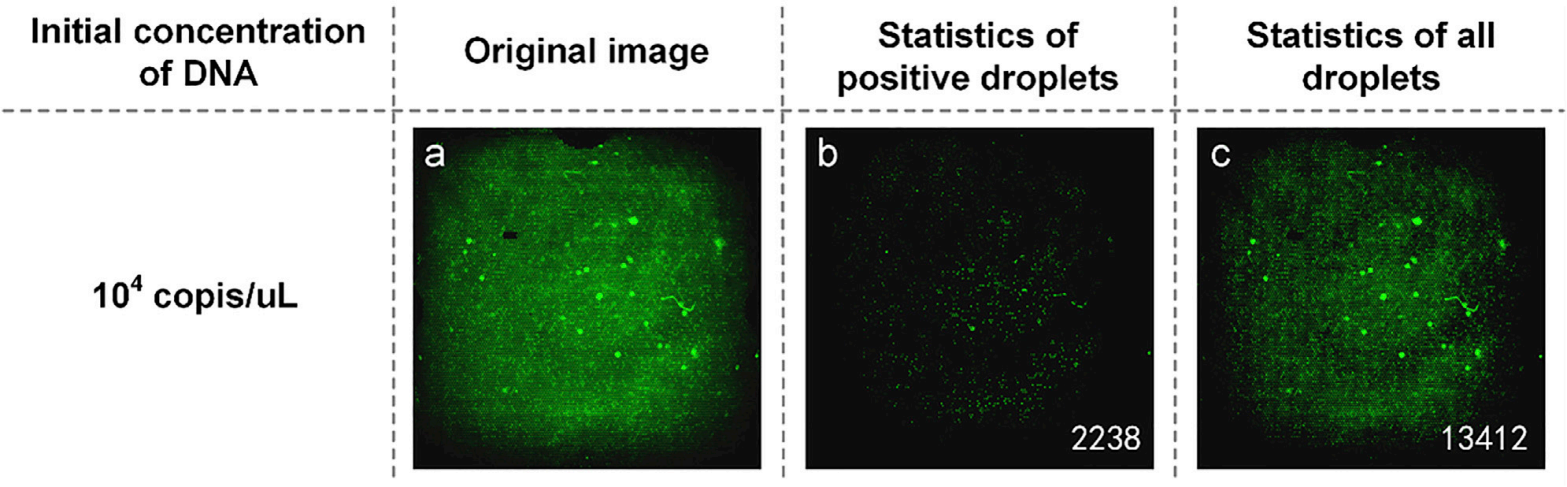
**(A)** Original fluorescence image of TP53 DNA; **(B)** Image processed by software and the number of positive droplets; **(C)** Statistics of all droplets with TP53 DNA.

Another part of the reaction solution was amplified in the dPCR chip. The fluorescent image captured by the detection device is shown in Figure 3A. The brightness of the image was still uneven, and there was a difference between the center brightness and the edge brightness, which made it difficult to count the microdroplets. We used MATLAB to write the image processing program, which was used to distinguish the original image’s positive droplets from the negative droplets (Figures 3B and C). The number of positive droplets was 2,238, and the number of all droplets was 13,412. This proves that our method is available.

The detection of a kind of DNA only showed that our method can quantify DNA by dPCR chip, without using a specific dPCR system. We also wanted to prove that such a combination could be used to detect reagents that could not be detected by commercial dPCR systems, breaking the detection limitations.

**FIGURE 4:**
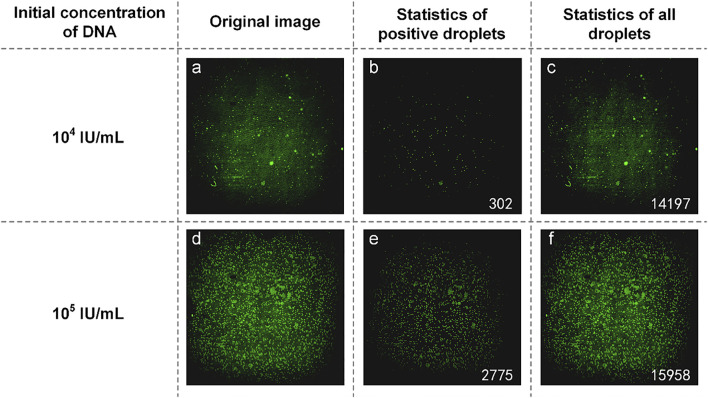
**(A)** Original image of HBV DNA with an initial concentration of 10^4^ IU/ml; **(B)** Image processed by software and the number of positive droplets with low-concentration DNA; **(C)** Statistics of all droplets containing HBV DNA with an initial concentration of 10^4^ IU/ml; **(D)** Original image of HBV DNA with a concentration of 10^5^ IU/ml; **(E)** Image processed by software and the number of positive droplets with high-concentration DNA; **(F)** Statistics of all droplets containing HBV DNA with an initial concentration of 10^5^ IU/ml.

The mixture of reaction solution containing HBV DNA could not be added to the reagents used in commercial dPCR, resulting in the concentration of HBV DNA not being detected by commercial dPCR. Therefore, we used the concentration ratio of the reaction liquid mixture itself to verify the results. In the experiment, DNA with an initial concentration of 1:10 was selected to prepare the mixture of the reaction solution. The experimental data are shown in Figure 4. From the original image (Figures 4A and D), the proportion of the number of positive droplets corresponding to the two initial concentrations of DNA was close to 1:10 by eyes. After analyzing the image, the accurate number of positive droplets and the total number of droplets were obtained. The number of positive droplets containing HBV DNA with an initial concentration of 10^4^ IU/ml is 302 (Figure 4B), and the number of all droplets containing HBV DNA with an initial concentration of 10^4^ IU/ml is 14,197 (Figure 4C). The concentration of DNA in the mixture of the reaction solution was 21.9404 copies/µl by using the quantitative formula of dPCR. the number of positive droplets containing HBV DNA with an initial concentration of 10^5^ IU/ml is 2,775 (Figure 4E), and the number of all droplets containing HBV DNA with an initial concentration of 10^5^ IU/ml is 15,958 (Figure 4F). The concentration of DNA in the mixture was 194.9308 copies/µl. The results showed that the ratio of DNA concentration calculated was close to 1:10, and the initial DNA concentration was 1:10. In consideration of the errors caused by the imprecise volume of each component in the configuration process of the reaction liquid mixture, as well as the errors produced in the sample loading process of the mixture, the test results are accurate.

**FIGURE 5:**
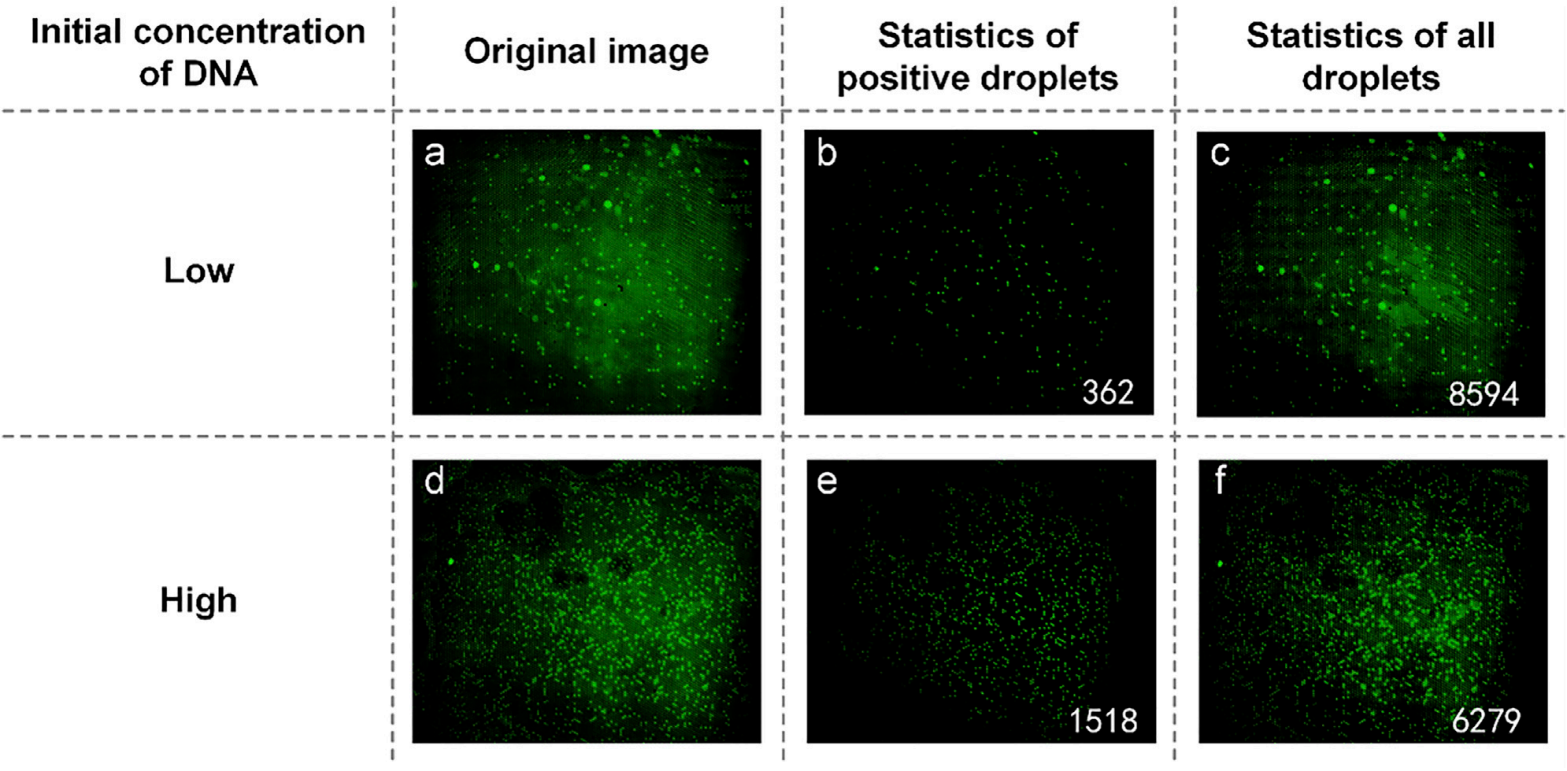
**(A)** Original image of UPE DNA with low concentration; **(B)** Image processed by software and the number of positive droplets with low-concentration DNA; **(C)** Statistics of all droplets containing UPE DNA with low concentration; **(D)** Original image of UPE DNA with high concentration; **(E)** Image processed by software and the number of positive droplets with high-concentration DNA; **(F)** Statistics of all droplets containing UPE DNA with high concentration.

UPE DNA was provided without explicitly indicating the initial DNA concentration. Here, it is expressed as high and low concentrations, and low concentration DNA was obtained by diluting the high concentration DNA 10-fold. Therefore, the relationship between the initial calculated DNA concentration of about 10 times proves the success of the experiment. Figures 5A and D are initial fluorescence images of UPE DNA. After processing and analyzing the image, we found that the number of positive droplets containing UPE DNA with low concentration (Figure 5B) was 362, and the number of all droplets containing UPE DNA with low concentration (Figure 5C) was 8,594. The concentration of DNA was 43.9136 copies/µl. Figures 5E and F show that the number of positive droplets containing UPE DNA with high concentration was 1,518, and the number of all droplets containing UPE DNA with high concentration was 6,279. After calculation, the concentration of DNA was 282.4010 copies/µl. The concentration of the two reaction mixtures was approximately 1:7, so for UPE DNA, our system could detect the presence of DNA, but the accuracy of the concentration calculation was not high enough.

**FIGURE 6:**
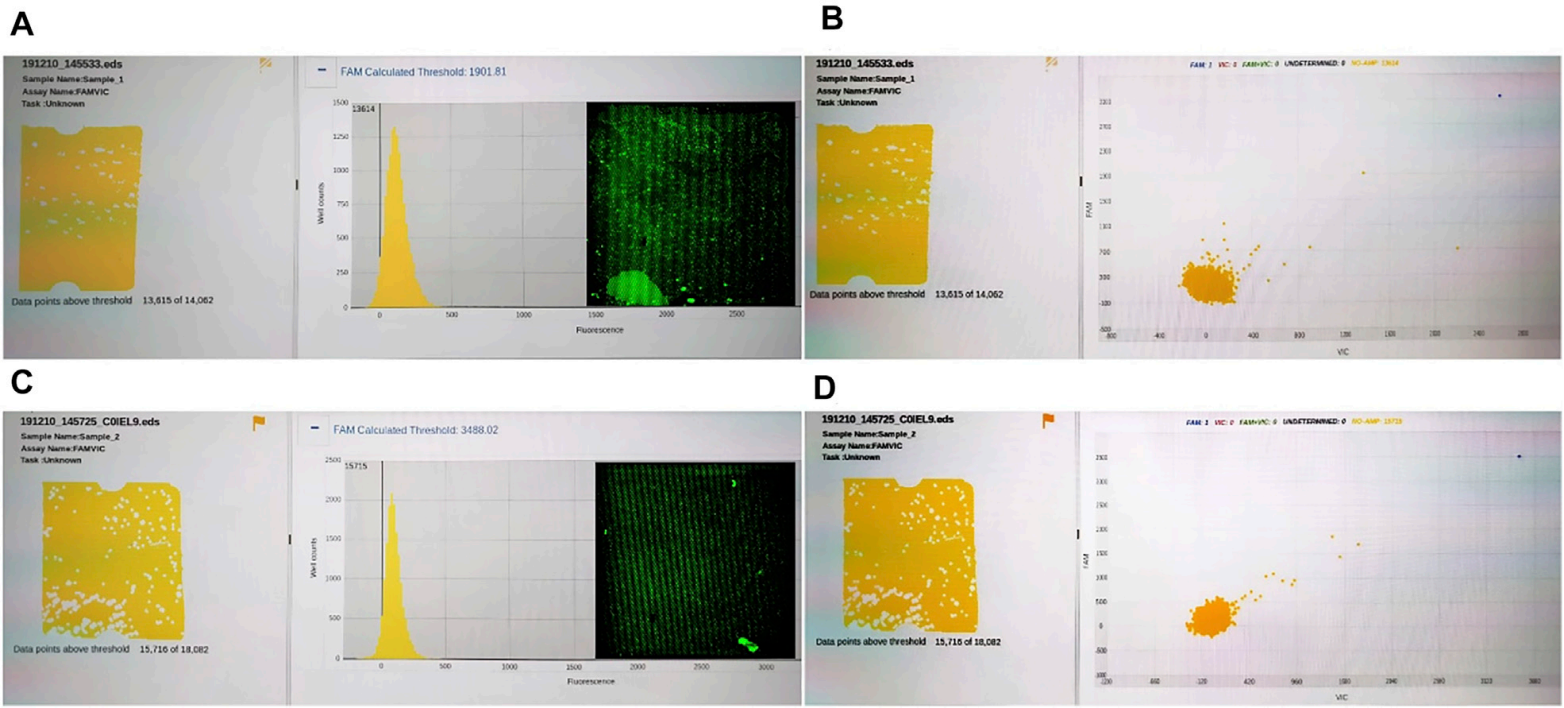
**(A)** Fluorescence intensity distribution of HBV DNA; **(B)** Fluorescence intensity distribution of HBV DNA; **(C)** Fluorescence intensity distribution of UPE DNA; **(D)** Fluorescence intensity distribution of UPE DNA.

To compare the universality of reagents between our system and commercial instruments, we compared the results of QuantStudio 3D digital PCR system and RainSure digital PCR system. The results showed that RainSure digital PCR system could detect TP53 DNA matched with it (picture 2), but it could not get the detection results of HBV DNA. In addition, for QuantStudio 3D digital PCR system, 20 µl of reaction solution containing HBV DNA and UPE DNA, respectively, in the abovementioned experiments was reserved, respectively. The reaction solution was coated in the dPCR chip according to the operating instructions. The chip was placed in the corresponding dPCR system for reaction and detection. Figure 6 shows that no amplification signal of dPCR chip was detected. Among them, Figures 6A and B are the detection results of the dPCR chip containing HBV DNA. Figures 6C and D are the detection results of the dPCR chip containing UPE DNA. The droplets in the detection results were all negative; the reaction solution did not contain the target DNA. Then, the chips were taken out from the detection device and placed in our detection system to observe the fluorescence. In the results, we observed not only the negative drops but also the positive ones. These images were added to Figures 6A and C, and the DNA in the results corresponds to Figures 6A and C, respectively.

**FIGURE 7:**
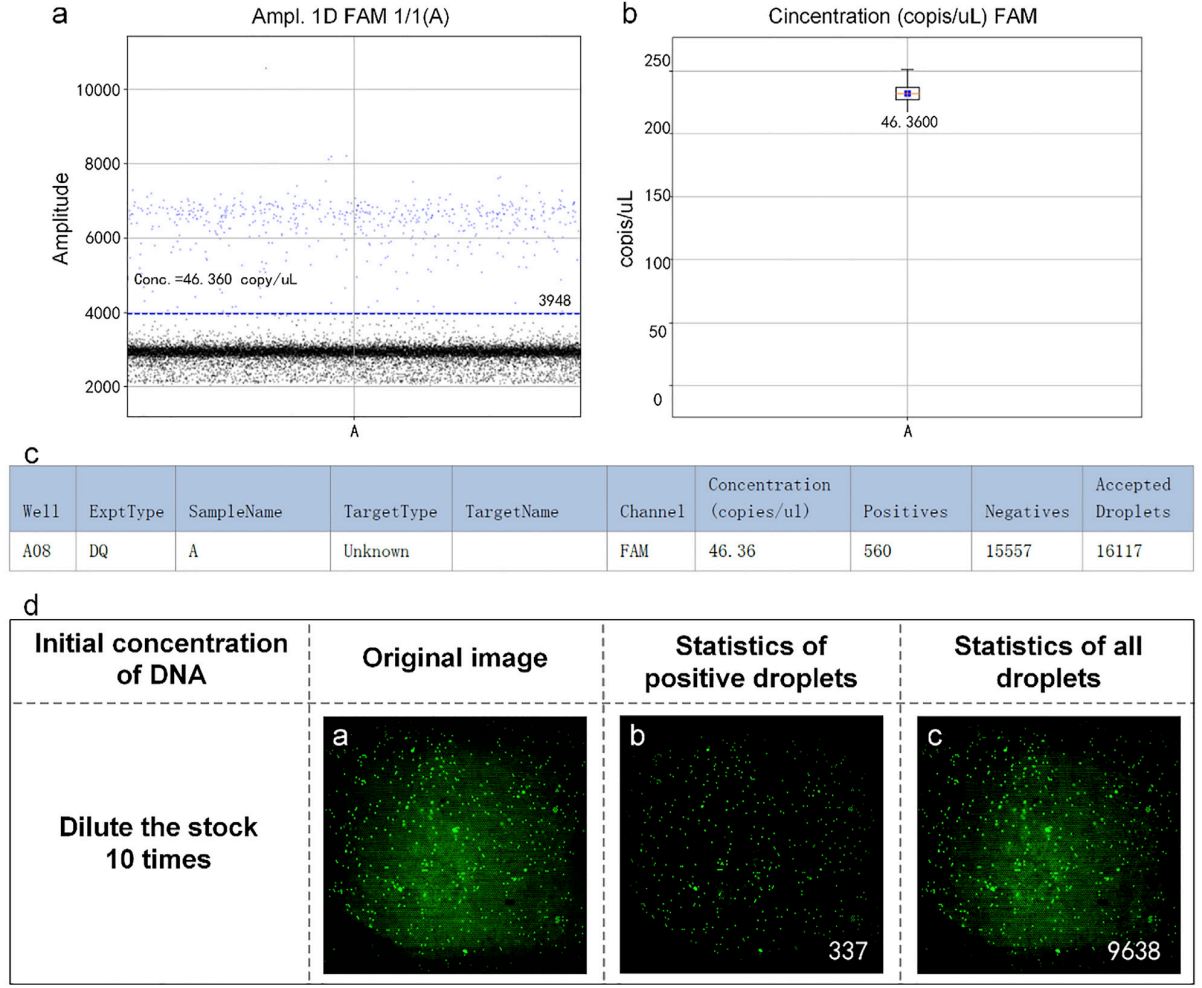
**(A)** Scatter plot by RainSure dPCR; **(B)** Initial DNA concentration calculated by RainSure dPCR; **(C)** Microdroplet data derived by RainSure dPCR; **(D)** Test results obtained with our system.

The reaction mixtures containing the leukemia sample were divided into two portions. One served as the control group detected by the RainSure dPCR system, while the other was spread on the dPCR chip as an experimental group and placed in a planar PCR instrument for amplification. The scatter plot given by the commercial instrument showed that the demarcation line of the fluorescence brightness of the negative droplet from the positive droplet was obvious (Figure 7A), and the sample concentration was 46.36 copies/µl (Figure 7B), of which, the number of positive droplets was 560, and the total number of droplets was 16,117 (Figure 7C). Figure 7D illustrates the test results given by our system: the number of positive droplets is 337, and the total number of droplets is 9,975. The measured sample concentration was calculated to be 42.96 copies/µl.

In early 2020, COVID-19 swept the world. Therefore, we performed the test for the detection ability of the SARS-COV-2 sample. Similar to HBV DNA, the mixture of reaction solutions that we used containing the SARS-COV-2 sample was also not detectable by the RainSure dPCR system. Therefore, the validation method is the same as that for HBV DNA. Samples with an initial concentration difference of 10 times were selected for detection.

**FIGURE 8:**
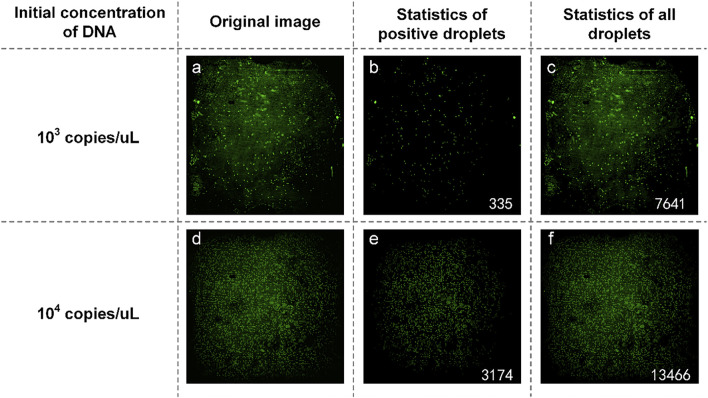
**(A)** Original image of SARS-COV-2 DNA with a concentration of 10^3^ copies/µl; **(B)** Image processed by software and the number of positive droplets with a concentration of 10^3^ copies/µl; **(C)** Statistics of all droplets containing SARS-COV-2 with a concentration of 10^3^ copies/µl; **(D)** Original image of SARS-COV-2 DNA with a concentration of 10^4^ copies/µl; **(E)** Image processed by software and the number of positive droplets with a concentration of 10^4^ copies/µl; **(F)** Statistics of all droplets containing SARS-COV-2 DNA with a concentration of 10^4^ copies/µl.

The plasmid containing SARS-COV-2 DNA was tested first. In the experiment, SARS-COV-2 DNA at an initial concentration of 1:10 was selected to prepare the reaction solution mixture. They are spread on dPCR chips and placed in a planar PCR instrument for amplification. As shown in Figure 8, the number of positive droplets of the reaction solution mixture with an initial concentration of 10^3^ copies/µl was 335 (Figure 8B), the total number of droplets was 7,976 (Figure 8C), and the sample concentration was 53.64 copies/µl. The number of positive droplets of the reaction solution mixture with the initial concentration of 10^4^ copies/µl was 3,174 (Figure 8E), the total amount of droplets was 16,640 (Figure 8F), and the sample concentration was 264.55 copies/µl. The two concentrations differed numerically by an order of magnitude, so the detection was successful.

**FIGURE 9:**
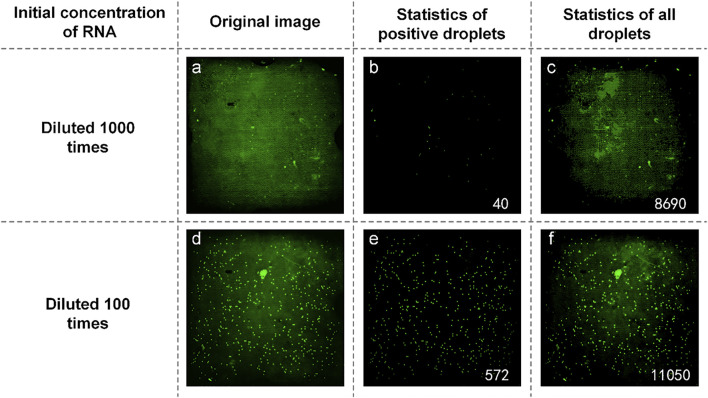
**(A)** Original image of SARS-COV-2 RNA with a concentration of diluted 1,000 times; **(B)** Image processed by software and the number of positive droplets with a concentration of diluted 1,000 times; **(C)** Statistics of all droplets containing SARS-COV-2 RNA with a concentration of diluted 1,000 times; **(D)** Original image of SARS-COV-2 RNA with a concentration of diluted 100 times; **(E)** Image processed by software and the number of positive droplets with a concentration of diluted 100 times; **(F)** Statistics of all droplets containing SARS-COV-2 RNA with a concentration of diluted 100 times.

We also obtained samples containing SARS-COV-2 RNA from the Wuhan Institute of Virology and prepared a reaction mixture with an initial concentration ratio of 1:10. They were spread on dPCR chips and placed in a planar PCR instrument for amplification. As shown in Figure 9, the number of positive droplets of the reaction solution mixture added with the sample diluted 1000-fold was 40 (Figure 9B), the total number of droplets was 4,730 (Figure 9C), and the sample concentration was 10.62 copies/µl. The number of positive droplets of the reaction mixture of samples diluted 100-fold was 572 (Figure 9E), the total number of droplets was 11,622 (Figure 9F), and the sample concentration was 63.09 copies/µl. The two concentrations differ by an order of magnitude numerically, so our system can quantitatively detect SARS-COV-2.

## Conclusion

In this study, the commercial dedicated dPCR chip is combined with the cheaper traditional planar PCR instrument and the fluorescence detection system built by the team to build a dPCR system with a lower price and wider application range. This makes the dPCR chip out of the dedicated system. Under the condition that DNA can be amplified normally, dPCR function can be realized with simpler equipment by using traditional planar PCR apparatus to provide thermal cycling conditions. The experiment proved that traditional planar PCR can provide suitable reaction conditions for dPCR chips. Under the detection system, the amplified microdroplets can be clearly distinguished, and the detection results are accurate, which is consistent with those of commercial PCR systems. During the experiment, we also found that, for commercial dPCR systems, some DNA cannot be detected due to the special formulation and reagents (such as HBV DNA, UPE DNA, and SARS-COV-2 sample) when they are configured as reaction solutions. But the fluorescence signal of such DNA can be clearly detected by our system, and the concentration can be calculated. In addition, we used TEC to build our own thermal cycling device, and the dPCR chips were used for testing. Experiments showed that the DNA in the chip can be amplified under this thermal cycling device, and the result was accurate. In general, our system used cheaper and simpler equipment to realize the functions of expensive and complex commercial dPCR systems. Moreover, our system has a wider detection range than commercial dPCR systems (RainSure dPCR system).

## Data Availability

The original contributions presented in the study are included in the article/Supplementary Material; further inquiries can be directed to the corresponding author.
